# Managing the “Sword of Damocles” of Immunosuppression: Prevention, Early Diagnosis, and Treatment of Infectious Diseases in Kidney Transplantation

**DOI:** 10.3390/pathogens12050649

**Published:** 2023-04-27

**Authors:** Roberto Cacciola, Serena Delbue

**Affiliations:** 1 Department of Surgery, King Salman Armed Forces Hospital, Tabuk 47512, Saudi Arabia; 2 Department of Surgical Sciences, University of Tor Vergata, 00133 Rome, Italy; 3 Biomedical, Surgical and Dental Sciences, University of Milan, 20122 Milano, Italy; serena.delbue@unimi.it

The careful tailoring of the most appropriate immunosuppressive strategy for recipients of a kidney transplant (KT) regularly faces a risk of complications that may harm the actual graft and affect patient survival. Balancing the benefits of effective induction immunosuppression followed by adequate maintenance of immunosuppressive therapy with the risks posed by infectious complications requires remarkable awareness, as well as a proactive approach to preventing such complications, or in fact an early diagnosis that may mitigate their invasiveness.

All articles of the Special Issue entitled “Infectious Complications in Chronic Kidney Disease and Renal Transplant Patients: Prevention, Diagnosis, Management, and Emerging Trends”, originating from a wide platform of transplant professionals, indicate without compromise that “prevention is better than cure”. The phrase, generally attributed to Desiderius Erasmus in the 16th century, and then more consistently recognised as a statement of Benardino Ramazzini [1], embodies the spirit of modern healthcare to the extent of being the actual motto of many advanced healthcare systems [2]. The increased vulnerability of recipients of solid organ transplant (SOT) to infectious complications unequivocally leads to the adoption of the very same principle in the daily care, as well as in the scientific advancements we have achieved in recent years.

The successes with the utilization of organs from extended criteria donors (ECD) and donors after circulatory death (DCD) represent a remarkable advancement for patients awaiting the allocation of a kidney from a deceased donor. In fact, the time spent on the transplant waiting list associated with the risk of suspension remains a major cause of mortality and reduced post-transplant survival for transplant patient [3]. The implementation of safe strategies aims to reduce the negative impact of delayed graft functions (DGF) on length of hospitalisation, as indicated by the enhanced recovery after surgery (ERAS) pathway, reflecting the evolution of current practice in the management of KT recipients [4]. Merging the benefits of reducing the impact of hospital-acquired infections with a cautiously tailored decision process on early biopsy in the context of DGF [5] represents a major clinical benefit for KT recipients together with being a cost-saving resource for healthcare providers and wider healthcare services [6].

The application of careful risk stratification in combination with the evaluation of defined biochemical parameters allows the clinical relevance of Cytomegalovirus Disease (CMVd), which remains a highly prevalent opportunistic viral infection in the first year following KT, to be assessed. Although it may not affect early graft or patient survival, as suggested in the series presented by Alfieri et al. [7], the prompt diagnosis, assessment, and treatment, where appropriate, can undoubtedly help to mitigate the negative impact of CMVd.

Infectious complications observed less frequently in the general population, represent a real “Sword of Damocles” hanging over patient survival following a KT. Among the others, two serious infections deserve particular attention. The occurrence of cryptococcosis, in the form of cryptococcal meningitis, represents a major threat to the recipients of a KT. The extensive national overview provided by Tardieu et al. [8] offers a valuable perspective on this highly critical condition. In the study, which documents nationwide disease transmission of two decades, the concepts of a high level of suspicion, early diagnosis, and prompt treatment represent pillars for the management of an infectious disease associated with a high mortality.

Similarly, the study of Tamzali et al. [9] contributes to raising our awareness of infectious endocarditis, which is associated with a diminished recipient and graft survival. The elegantly designed study once more supports the notion that immunosuppressive treatment associated with multiple comorbidities is a major risk factor for the development of severe infectious complications.

The ongoing SARS-CoV-2 pandemic has forced the transplant community, our patients, and their families to make highly complex decisions [10,11], particularly at a time when non-pharmaceutical interventions were the only measure available to protect the most vulnerable and immunosuppressed patients [12]. The impact of COVID-19 in the geographical “epicentre” of the pandemic is clearly presented by Perego et al. [13]. Dedicated personnel and patient pathways, together with the relentless application of preventive measures, have allowed the viability of the KT services to be maintained. Undoubtedly, the contrary would have further compromised the survival of patients with end-stage renal disease (ESRD). Campise et al. [14] report a relatively low incidence of COVID-19 infection among KT recipients associated with a higher mortality compared to the general population. Certainly, the numerous immunosuppressive strategies tailored around specific risk factors have helped to mitigate the impact of a novel and unknown virus on the recipients of a KT [15].

Preventing infectious diseases following KT remains one of the unavoidable challenging aspects of current practice, particularly when addressing high-risk recipients of any SOT. Reducing the overall immunosuppressive dose requires a careful balance; while in liver transplantation it appears to be a realistic objective [16], recent advancements in KT indicate that the induction of tolerance through chimerism may very soon reach the stage of human trials following successful outcomes in other animals [17]. Beyond the fast acquisition of knowledge on aspects of molecular biology that may lead to a reduced risk of infectious diseases, actual risk elimination may be achieved through bio-engineering and artificial organs [18]. Undoubtedly, the advancing technology of wearable or implantable devices represents the most promising innovation for the future treatment of patients with ESRD [19].
